# A novel taxonomic database for eukaryotic mitochondrial cytochrome oxidase subunit I gene (eKOI), with a focus on protists diversity

**DOI:** 10.1093/database/baaf057

**Published:** 2025-09-24

**Authors:** Rubén González-Miguéns, Àlex Gàlvez-Morante, Margarita Skamnelou, Meritxell Antó, Elena Casacuberta, Daniel J Richter, Enrique Lara, Daniel Vaulot, Javier del Campo, Iñaki Ruiz-Trillo

**Affiliations:** Institut de Biologia Evolutiva (CSIC-Universitat Pompeu Fabra), 08003 Barcelona, Spain; Institut de Biologia Evolutiva (CSIC-Universitat Pompeu Fabra), 08003 Barcelona, Spain; Institut de Biologia Evolutiva (CSIC-Universitat Pompeu Fabra), 08003 Barcelona, Spain; Institut de Biologia Evolutiva (CSIC-Universitat Pompeu Fabra), 08003 Barcelona, Spain; Institut de Biologia Evolutiva (CSIC-Universitat Pompeu Fabra), 08003 Barcelona, Spain; Institut de Biologia Evolutiva (CSIC-Universitat Pompeu Fabra), 08003 Barcelona, Spain; Real Jardín Botánico de Madrid (RJB-CSIC), 28014 Madrid, Spain; Sorbonne Université, CNRS, UMR7144, Station Biologique de Roscoff, 29680 Roscoff, France; Department of Biosciences, University of Oslo, PO Box 1066 Blindern, 0316 Oslo, Norway; Institut de Biologia Evolutiva (CSIC-Universitat Pompeu Fabra), 08003 Barcelona, Spain; Institut de Biologia Evolutiva (CSIC-Universitat Pompeu Fabra), 08003 Barcelona, Spain; ICREA, 08010 Barcelona, Spain

## Abstract

Metabarcoding has emerged as a robust method for assessing biodiversity patterns by retrieving environmental DNA directly from ecosystems. While the 18S rRNA gene is the primary genetic marker used for broad eukaryotic metabarcoding, it has limitations in resolving lower taxonomic levels. A potential alternative is the mitochondrial cytochrome oxidase subunit I (COI) gene because it offers resolution at the species level. However, the COI gene lacks a comprehensive, curated taxonomically informed database including protists. To address this gap, we introduce eKOI, a novel, curated COI gene database designed to enhance the taxonomic annotation for protists that can be used for COI-based metabarcoding. eKOI integrates data from GenBank and mitochondrial genomes, followed by extensive manual curation to eliminate redundancies and contaminants, recovering 15 947 sequences within 80 eukaryotic phyla. We validated the use of eKOI by reannotating several COI metabarcoding datasets, revealing previously unidentified protist biodiversity and demonstrating the database utility for community-level analyses.

## Introduction

Metabarcoding has emerged as a powerful tool in the last two decades [1], allowing researchers to comprehend biodiversity patterns without the biases of traditional sampling methods [2]. Under this approach, DNA is directly retrieved from the environment (eDNA), allowing the characterization of microbial communities without the need for isolation or culture-dependent approaches [3,4]. Furthermore, its affordability nature has broadened application across diverse biological disciplines. For example, metabarcoding has provided novel insights into biogeographical [5,6] and ecological [7,8] patterns. However, the success of metabarcoding hinges on well-curated and comprehensive reference taxonomic databases to accurately annotate the sequenced eDNA.

Ribosomal genes, particularly the 18S rRNA gene (18S), are the most widely used genetic markers for species delimitation and phylogenetic inference within eukaryotes, including protists. Their widespread use is due to their universality (they are present in all living beings), the presence of both conserved and hypervariable regions that facilitate a phylogenetic resolution at various taxonomic levels, and the availability of different generalist and taxon-specific primer sets. As a result, several large taxonomic databases have been generated, such as PR^2^ [9] or SILVA [10], which are essential for accurate taxonomic annotation in metabarcoding studies.

However, the 18S has limitations in resolving taxonomy at the intraspecies levels, due to its highly conserved nature [11,12]. To overcome this limitation, researchers have explored more divergent noncoding regions within and between ribosomal genes, such as the internal transcribed spacer [13], or protein-coding genes like the ribulose-bisphosphate carboxylase gene (rbcL) [14,15]. While these alternative markers offer improved taxonomic resolution appropriate for species delimitation, taxonomic databases for these markers often exhibit bias towards specific groups, such as fungi or diatoms [16,17]; although recent databases like EUKARYOME [18] aim to cover the full breadth of eukaryotic diversity.

The mitochondrial cytochrome oxidase subunit I (COI) gene has been proposed as the barcode gene for species delimitation in metazoans [19]. The COI gene has also started to be applied to the taxonomy and systematics of protists, including groups such as testate amoebae [20,21], foraminifera [22], coccolithophores [23], and diatoms [24]. This has led to the creation of several reference COI taxonomic databases, like BOLD [25], CO-ARBitrator [26], and MIDORI2 [27]. However, these existing databases are predominantly biased towards metazoans, and often lack curated and nonredundant sequences for other eukaryotic groups. Additionally, they may lack standardized taxonomic ranks that are commonly used for eukaryotes [28]. These limitations hinder the effective application of the COI gene in community-level studies of eukaryotes, particularly those including protists, and obstruct taxonomic integration across different molecular marker databases, such as the widely used PR^2^ for 18S rRNA gene [9].

To address these limitations, we introduce eKOI (environmental eukaryotic cytochrome oxidase subunit I) (version 1.0), a novel and curated eukaryote-wide database encompassing 80 phyla of the mitochondrial COI gene. This new reference database aims to overcome the limitations of existing COI taxonomic databases at the protist community-level, analogous to what PR^2^ represents for 18S. This will facilitate accurate taxonomic annotation of metabarcoding sequences, as well as comparisons among different taxonomic databases derived from other molecular markers. To create this dataset, we combined COI gene data extracted from GenBank and the complete COI gene obtained from publicly available mitochondrial genomes. A thorough and manual curation process was implemented to eliminate redundant sequences, identify potential contaminants, and correct taxonomic annotation errors. We evaluated eKOI by taxonomically reannotating various COI-based metabarcoding studies, resulting in the identification of previously unidentified diversity. Finally, we further validated the new taxonomic annotations for these studies by constructing phylogenetic trees using sequences from the eKOI database, thereby confirming the large amount of protist biodiversity previously uncharacterized with the COI gene.

## Materials and methods

### GenBank database sequences downloading and curation

To construct the eKOI taxonomic database, we initially retrieved the sequences from the mitochondrial gene ‘Cytochrome oxidase subunit I’ (COI) from GenBank (Fig. S1). We established keywords to search for each taxonomic group in the ‘NCBI taxonomy browser’: ‘((“X”[Organism] OR “X”[All Fields]) AND co1[All Fields]) OR ((“X”[Organism] OR “X”[All Fields]) AND cox1[All Fields]) OR ((“X”[Organism] OR X[All Fields]) AND coi[All Fields]) OR ((“X”[Organism] OR X[All Fields]) AND cytochrome oxidase[All Fields] AND subunit[All Fields] AND 1[All Fields]) OR ((“X”[Organism] OR “X”[All Fields]) AND cytochrome oxidase[All Fields] AND subunit[All Fields]) OR ((“X”[Organism] OR “X”[All Fields]) AND coxi[All Fields])’, where ‘X’ denotes the name of each major taxonomic group of ‘NCBI taxonomic browser’ within ‘Eukaryota’. The files were downloaded in ‘INSDSeq XML’ format, obtaining about 4 million sequences. The resultant sequences, grouped by taxonomic groups, mainly phyla, were processed using a custom script 1_sequences_procesing.py. This script eliminated the sequences that were duplicated, smaller than 200 bp and larger than 3000 bp. Next, to reduce the redundancy and the total number of sequences, clusters were created based on similarity percentages using vsearch ver. 2.14.1 [29], selecting a representative sequence for each cluster. The similarity percentage was established at 97%, except for Arthropoda, Chordata, and Mollusca for which it was set at 90%, aiming to balance the number of sequences per taxonomic group in the final eKOI database. This limits identifications to low taxonomic levels such as species or genus. Chimeric sequence detection was performed using vsearch ver. 2.14.1 ‘*de novo*’ [29] per phylum and the final database, using default settings. Lastly, a ‘fasta’ file was generated for each taxonomic group containing the sequences with the taxonomy string defined in GenBank.

Alignments were generated for each taxonomic group, using MAFFT version 7.490 [30], using default parameters. Finally, manual curation of the resulting sequences was performed using the software Geneious Prime (version 2019.0.4), removing divergent sequences that may be errors or taxonomic misclassifications.

### Mitochondrial genome database curation and integration with GenBank database

The resulting curated sequences retrieved from GenBank were combined with the mitochondrial genome of public databases, such as GenBank and Zenodo. The COI gene was extracted from complete mitochondrial genomes present in GenBank based on the sequence annotations. Some resulting sequences contained exons and introns. It has been demonstrated that some introns can result from nuclear pseudogenes [31]. Therefore, we first tested whether the introns were potential pseudogenes or chimeric sequences. Additionally, the presence of pseudogenes was confirmed through alignments, verifying whether there were divergent sequences. To accomplish this, two datasets were generated from the mitochondrial genomes: one with the entire COI gene including introns and exons, and another one containing only the coding region, the exons.

Alignments were performed using MAFFT version 7.490 [30], using default parameters, aiming to determine if the sequences from the GenBank database or the newly amplified sequences contained the intron region. The script 2_percentage_identity_graphic.py graphically represents the percentage of identity for each position in an alignment. Once the introns were confirmed to be potential pseudogenes or contaminations, the curated GenBank sequences were combined with the coding regions of the COI gene extracted from the mitochondrial genomes (see results section ‘Testing the presence of introns within COI gene’). The final fasta files and alignments for each taxonomic group are available from the Supplementary data. Finally, the alignments without introns were used to curate the sequences with a wrong taxonomy following the ‘curation process’ in EukRef [32], generating phylogenetic reference trees and alignments per phylum.

### Taxonomy path curation

One of the limitations of current molecular databases for the taxonomic annotation of eDNA sequences is how to handle the variable taxonomic ranks across clades of eukaryotes. Even though higher taxonomic ranks, such as order or class, lack comparable evolutionary context, in terms of divergence time or evolutionary history in general, many computational tools require a fixed number of ranks across taxa.

To achieve this, the names of the sequences were extracted into a CSV file using the script 3_fasta_name_extraction.py. This file was manually curated to correct the taxonomy of each sequence. The aim was to ensure that all sequences have ‘homologous’ taxonomic categories among them at each taxonomic level. We used the taxonomic levels proposed by PR^2^ version 5.0 (released in 2023) [9], comprising nine levels: ‘domain; supergroup; division; subdivision; class; order; family; genus; species’. We also generated another dataset, to which we manually added the level ‘phylum’ between ‘subdivision’ and ‘class’ since phylum is one of the most widely used taxonomic ranks in metazoans. Once each CSV file was manually curated, the names of the sequences in the fasta file were substituted by the accession identifier unique to each sequence, using the script 4_fasta_name_substitution.py. The final eKOI taxonomic database version 1.0 is available from the supplementary data (file ‘eKOI_ver1.fasta’).

### Testing the eKOI database accuracy and comparing with other taxonomic databases

To test the accuracy of the eKOI database (version 1.0), 15 metabarcoding studies based on COI were selected (see Table 1 for details on metabarcoding studies and primers used). The raw data of these studies was reanalysed using the protocol described in [33], using the dada2 R package version 1.32 [34]. Subsequently, the resulting amplicon sequence variants (ASVs) smaller than 100 bp were discarded, as taxonomic annotations for fragments of such small size are typically unreliable.

**Table 1.** Metabarcoding studies based on the COI molecular marker that has been reanalyzed with our new eKOI dataset, together with the primers used in each case and the environment

ID	Paper	Primer R	Primer F	Environment
1	[57]	ArCOIR [20]	LCO [40]	Freshwater and soil
2	[33]	ArCOIR [20]	LCO [40]	Freshwater and marine
3	[58]	HCO [40]	LCO [40]	Soil
4	[44]	jgHCO2198 [41]	mlCOIintF-XT [43]	Marine
5	[59]	jgHCO2198 [41]	mlCOIintF [42]	Marine
6	[60]	jgHCO2198 [41]	mlCOIintF [42]	Marine
7	[46]	jgHCO2198 [41]	mlCOIintF [42]	Marine
8	[61]	jgHCO2198 [41]	mlCOIintF [42]	Freshwater
9	[62]	jgHCO2198 [41]	mlCOIintF [42]	Marine
10	[63]	jgHCO2198 [41]	mlCOIintF [42]	Freshwater
11	[64]	jgHCO2198 [41]	mlCOIintF [42]	Marine
12	[65]	EPTDr2n [66]	fwhF2 [67]	Freshwater
13	[68]	HCO [40]	LCO [40]	Freshwater
14	[69]	jgHCO2198 [41]	mlCOIintF [42]	Marine
15	[45]	jgHCO2198 [41]	mlCOIintF [42]	Marine

Taxonomic annotation was performed using the eKOI and MIDORI2 [27] databases (accessed 27 October 2024). For this purpose, a custom script 5_taxonomic_assignation.py was employed. This script generates an independent folder for each fasta file present in the script’s directory. Within each folder, an Excel file is generated containing the taxonomic annotation information for each ASV using vsearch usearch_global command version 2.14.1 [29]. ASVs with lower than 84% similarity to reference sequences were not considered. This threshold was established based on Amoebozoa from the order Arcellinida and metazoans COI pairwise distance [19,33]. However, this threshold should be applied with caution, as specific studies are needed to refine and validate it across eukaryotic phyla. Subsequently, a fasta file is generated for each desired taxonomic level. In this case, we selected phylum. The resulting ASVs taxonomically annotated for each phylum with eKOI can be downloaded from ‘3_ASV_metabarcoding’ in the supplementary data.

To graphically represent the diversity of each taxonomic group and test the accuracy of taxonomic annotations using eKOI, we generated phylogenetic trees for each taxonomic group, focusing on protists. To reduce the number of sequences for graphical representation, the ASVs were grouped into operational taxonomic units (OTUs) based on a similarity percentage of 97% using a custom script 6_cluster_OTU.py. Alignments of the resulting OTUs were performed using MAFFT version 7.490 [30], with default parameters, along with sequences from the eKOI database of the same taxonomic groups, and at least two sequences from sister groups as outgroups. Tree topologies and node supports were evaluated using maximum likelihood with IQTREE2 version 2.0, where the best substitution models were selected with ModelFinder [35]. Node supports were assessed with 10 000 ultrafast bootstrap replicate approximations [36]. The resulting trees were graphically edited using the R package ggtree version 3.12 [37].

For some groups (Apicomplexa, Cercozoa, Filasterea, and Heterolobosea), reconstructions of ancestral habitat characters were performed to illustrate the potential of the eKOI database. For that purpose, we used the phytools R package version 2.3 [38]. We employed the function *make.simmap* [39] to generate 500 stochastic character maps from our dataset, under the equal rates model. These sets of stochastic maps were then summarized using the function *densityMap*, plotting the posterior probability of being in each state across all the edges and nodes of the tree. For the graphical representation of the distribution maps of the new eDNA sequences, we used the maps R package version 3.4.2.

Once taxonomic annotations were made, the different samples were grouped by study (Table 1) and then by sampled environment. From these groups, the mean ASV taxonomic annotation values were obtained across all samples using the script 7_taxonomic_assignation_mean.py. This script generates a CSV with the percentage of the number of ASV assigned to each phylum. This was performed for the eKOI and MIDORI2 databases (‘5_COI_databses_comparison’ in supplementary data). Barplots were generated in R using ggplot2 version 3.5.1, selecting the phyla with at least 1% of ASV taxonomically assigned in each study.

## Results

### eKOI database

**Figure 1.**
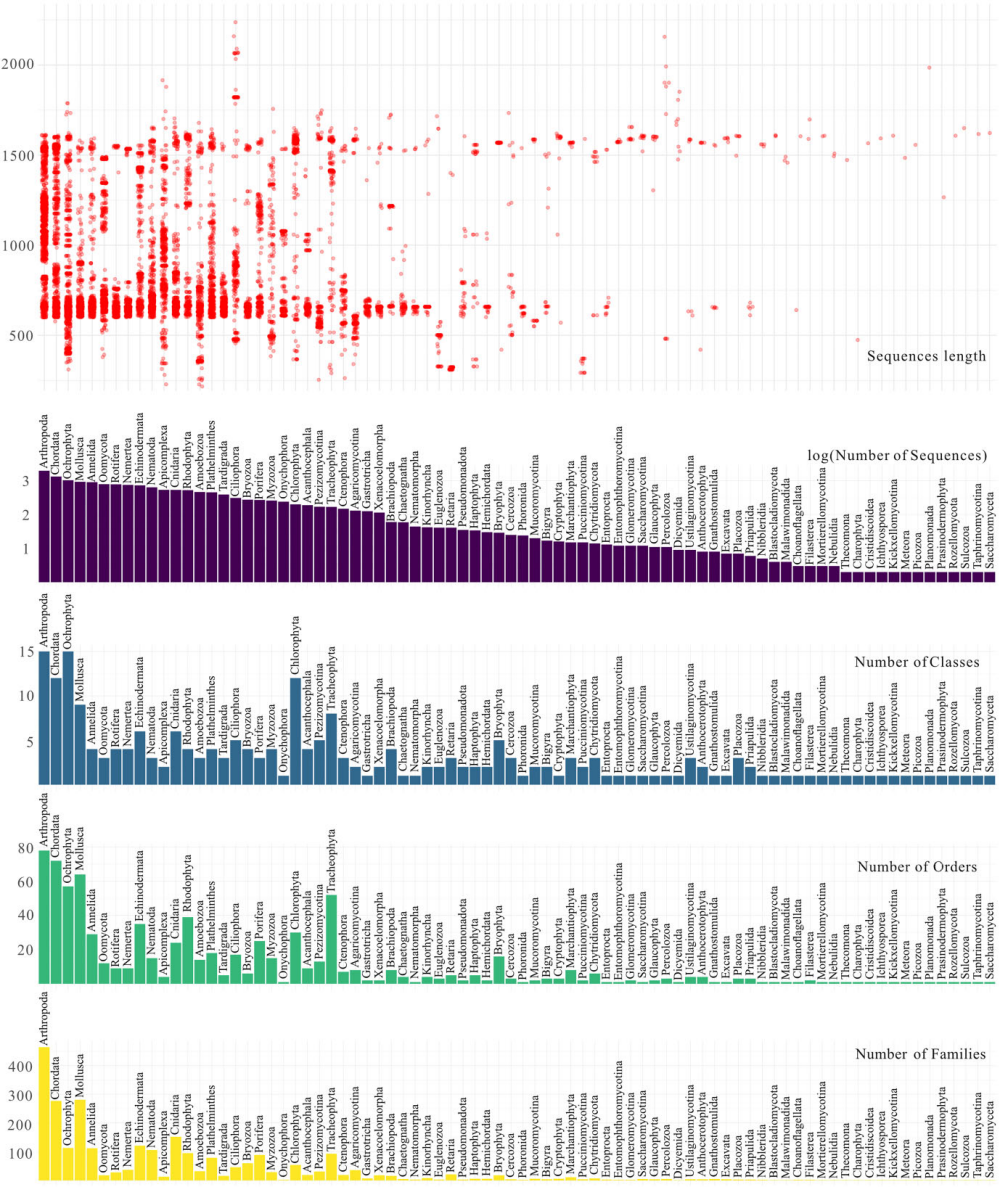
Graphs representing the number of families (yellow), orders (green), classes (blue), and total sequences (violet) per phylum present in the eKOI database. Red circles represent the size, in base pairs, of each sequence included in each phylum.

The final eKOI database includes 15 947 sequences, representing 80 eukaryotic phyla, 231 classes, 796 orders, and 2646 families of eukaryotes (Fig. 1 and ‘eKOI_taxonomy.csv’ in supplementary data). Therefore, eKOI expands protist taxonomic coverage compared to other COI databases and incorporates eukaryotic groups absent in existing databases, such as Picozoa, Nibbleridia, or Rozellomycota (see Tables S1 and S2 in supplementary data). Almost every eukaryotic phylum in the eKOI database is represented by at least one complete COI gene sequence. eKOI predominantly contains sequences of two lengths: ~600 base pairs and 1600 base pairs (Fig. 1). The ~600 bp sequences correspond to fragments amplified using the HCO and LCO primer pair [40], while the ~1600 bp sequences represent the complete COI gene obtained from mitochondrial genomes. The coding region (exons) of the COI gene exhibits relatively stable length across eukaryotic phyla.

To validate taxonomic annotations and the presence of introns, we constructed two alignments one with and one without introns (see the section ‘Materials and Methods’, ‘Mitochondrial Genome Database Curation and Integration with GenBank Database’). In the alignment including introns, some GenBank sequences displayed large gaps due to the absence of introns (Fig. S2). Sequences with introns were considered as potential pseudogenes and were removed [31]. The lack of highly divergent sequences and long branches in the phylogenetic trees, supports the taxonomy accuracy of the eKOI database (supplementary data folder ‘2_alignment_eKOI’).

### Applying the eKOI database in metabarcoding studies

**Figure 2.**
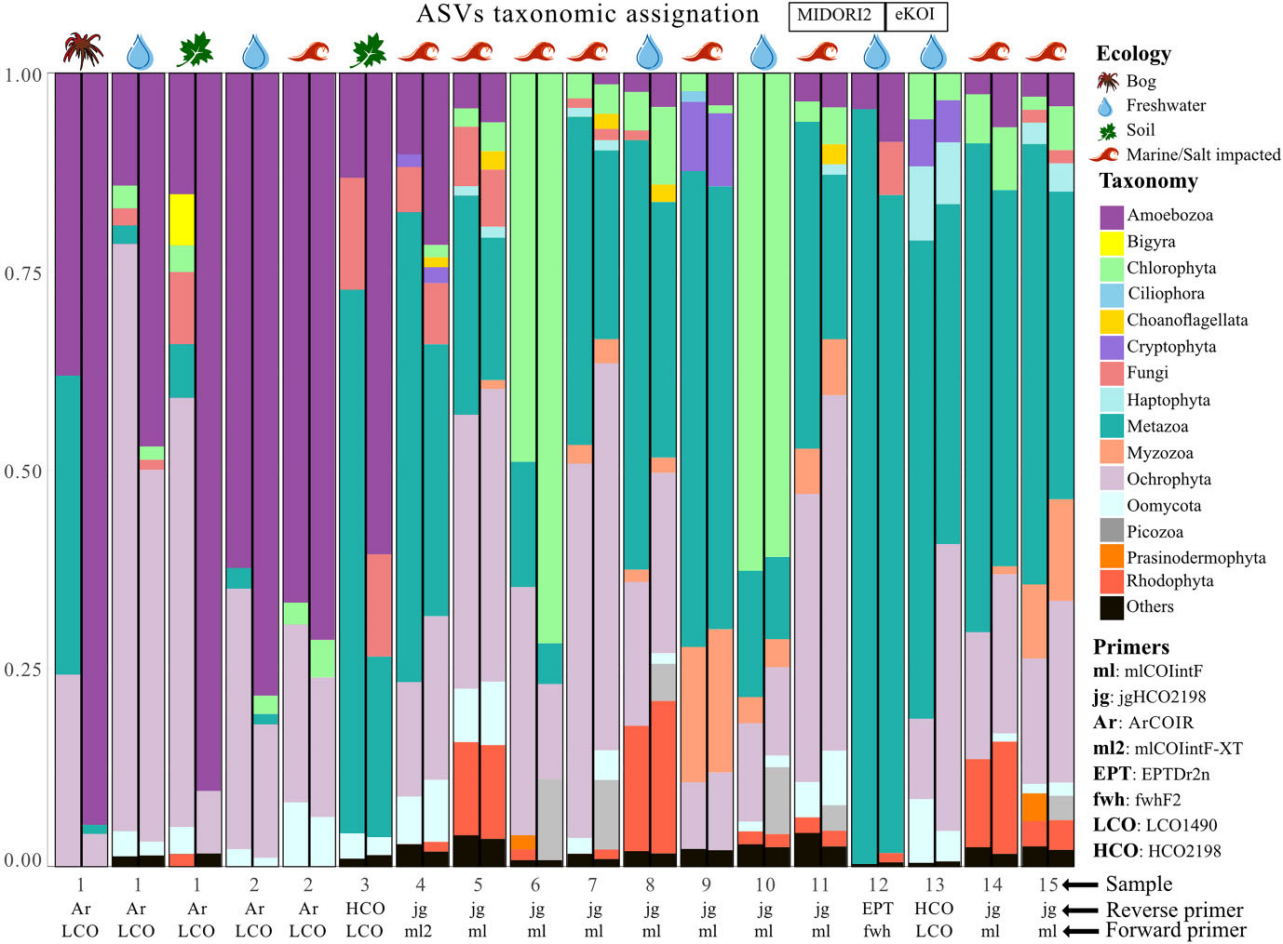
Bar chart representing the proportion of ASVs, relative to the total taxonomically classified, to each phylum in the MIDORI2 (left barplots) eKOI database (right barplots) from 15 metabarcoding studies (Table 1). Due to the large number of phyla present in each study, only the phyla with at least 1% of ratio per study are represented (the rest of the other phyla are represented as ‘others’). The metazoan phyla were grouped as ‘Metazoa’ and fungi phyla as ‘Fungi’. The environment type of each study is represented by different symbols. The pair of primers used in each study is also presented.

**Figure 3.**
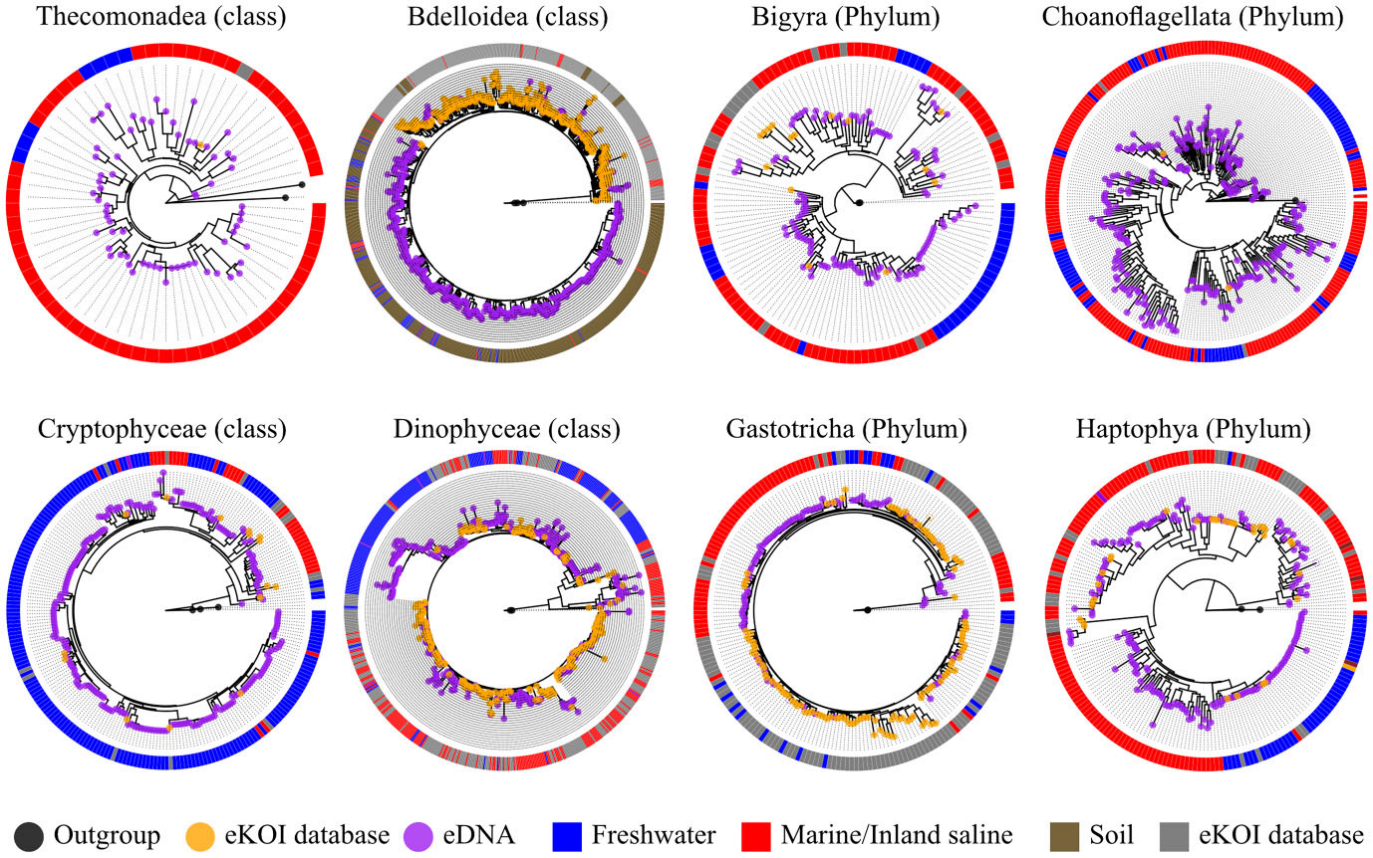
Phylogenetic trees obtained by combining sequences from the eKOI database of different taxonomic groups (orange dot), with outgroups (grey dot) and eDNA OTUs (purple dot). OTUs were obtained from similarity clustering of taxonomically reannotated ASVs from metabarcoding studies (Table 1). The circle surrounding each phylogenetic tree represents the environment of each OTU, sequences from the eKOI database are represented in grey, with the ecology not represented to reflect the newly characterized biodiversity for each taxonomic group.

To test the effectiveness and accuracy of the eKOI database to taxonomically assign eukaryotic COI eDNA diversity, we analysed 15 metabarcoding studies (Table 1 and Fig. 2). The prevalent use of primers jgHCO2198 [41], mlCOIintF [42], and mlCOIintF-XT [43] is due to their ability to amplify a diverse range of eukaryotic taxonomic groups. The variation in the proportion of phyla within similar ecosystem types, using identical primers, is linked to differences in sample substrates, such as plankton [44,45] versus sediments [46] for example. Across all studies, eKOI identified significant eukaryotic microbial diversity, including Amoebozoa, Chlorophyta, Choanoflagellata, and Picozoa, which constituted a substantial portion of ASVs (Fig. 2 and ‘eKOI_MIDORI2_comparison.xlsx’ in supplementary data). Notably, this diversity revealed a substantial underestimation of protist diversity and highlighted previously overlooked taxa, such as choanoflagellates and Picozoa (Fig. 3 and Fig. S3), which were not recovered using MIDORI2 (Fig. 2 and ‘eKOI_MIDORI2_comparison.xlsx’ in supplementary data). Instead, MIDORI2 database assigned a higher representation of metazoans, unlike eKOI (Fig. S4).

**Figure 4.**
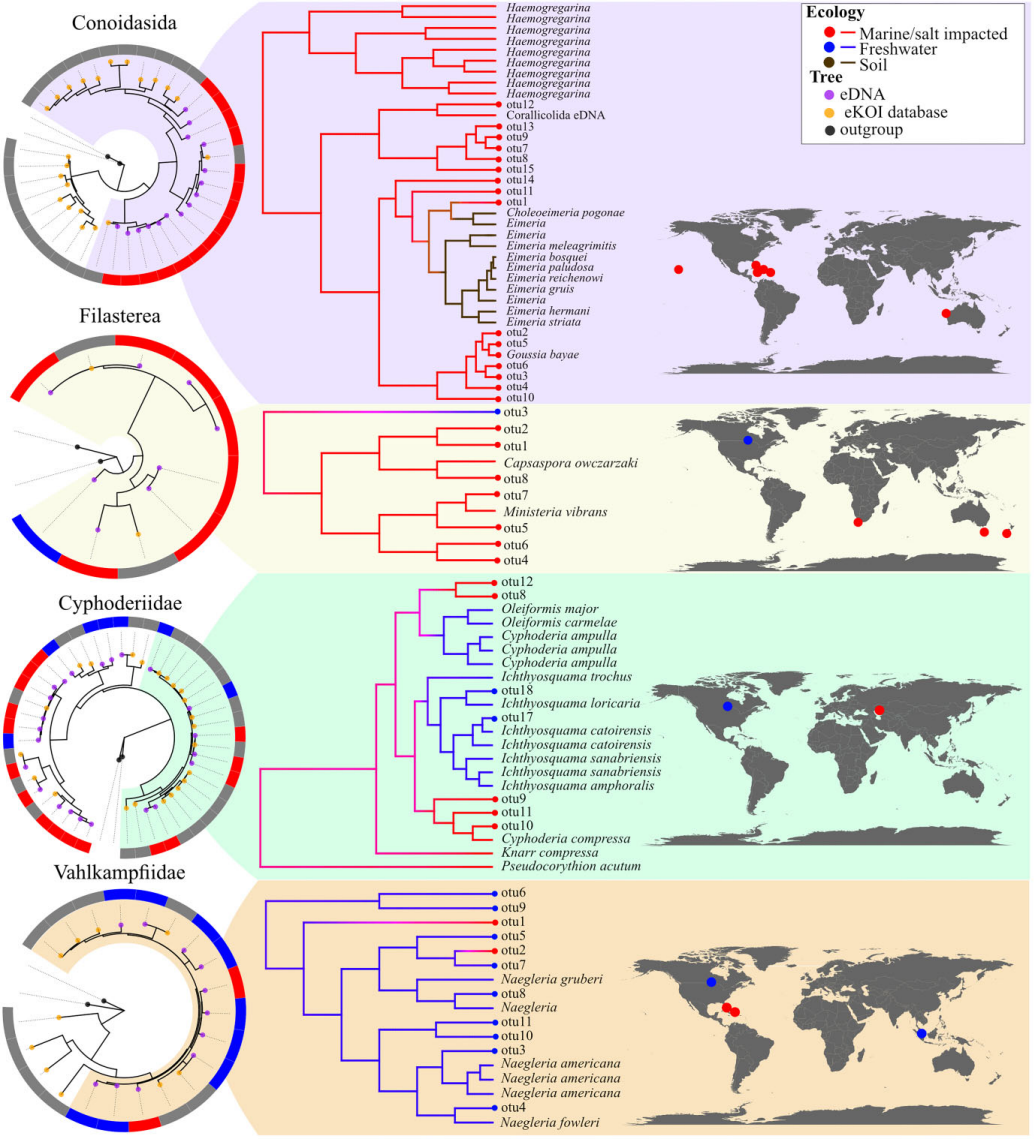
Phylogenetic trees obtained by combining sequences from the eKOI database of each taxonomic group (orange circles) and with outgroups (black circles). The eDNA OTUs (purple circles) were calculated and generated independently for each taxonomic group, with the IDs shared across groups. The circle surrounding each phylogenetic tree represents the habitat of each OTU, sequences from the eKOI database are represented in grey, to reflect the newly characterized biodiversity for each taxonomic group. From each taxonomic group, a part of the tree was selected from which the ancestral habitat was reconstructed, as an example. We also represented with dots on the map (blue for freshwater, brown for soil, and red for marine) the origin of the different OTUs.

The utility of ASVs derived from eDNA samples extends beyond characterizing novel molecular biodiversity. For instance, we examined the Cyphoderiidae (Rhizaria), a group in which ecological transitions across the salinity barrier have influenced its evolutionary history [47]. While these transitions were primarily characterized using the 18S marker, the COI database lacked marine lineages that were exclusively obtained with the nuclear marker. The integration of new COI ASVs (Table 1), taxonomically classified as Cyphoderiidae using the eKOI database, enabled the characterization of marine lineages that had been previously identified with 18S, like the ‘marine clade 1’ [47]. This increase in the Cyphoderiidae COI sequences enhances the robustness of characterizing ecological transitions between marine and freshwater environments (Fig. 4), allowing for consistent ecological and phylogenetic patterns using both nuclear and mitochondrial markers. This approach can be applied to other difficult-to-sample microorganisms, such as free-living or parasitic taxonomic groups; for example, Conoidasida (Apicomplexa) and Filasterea (Fig. 4).

## Discussion

The novel eKOI database fills a crucial taxonomic gap present for protist COI, allowing an integration of taxonomic annotation between 18S rRNA and COI metabarcoding studies. The inclusion of curated protist sequences mitigates certain limitations present in existing COI databases that primarily focus on metazoans. Reducing redundant sequences improves the application of eKOI in community-level studies of eukaryotes; however, this limits its utility for species-level assignments of metazoans. For these specific assignments, specialized databases such as BOLD [48] or MIDORI2 [27] and others focused on specific taxonomic groups, like insects [49], metazoans [26], or zooplankton [50] remain valuable for better taxonomic resolution within those clades.

The taxonomic curation and standardization of eKOI with databases such as PR^2^, which focuses on the 18S ribosomal gene, enables comparison of taxonomic annotation results. Currently, most community-level [51] or protist-focused [52] metabarcoding studies rely on 18S rRNA. However, a notable limitation of 18S rRNA is its phylogenetic resolution at lower taxonomic levels, due to its slow mutation rate compared to mitochondrial genes. While effective for inferring relationships at deeper taxonomic scales, its utility diminishes for species or intraspecific level distinctions [12,33], with some exceptions [53,54]. To solve this problem, fast-evolving genes such as COI provide species-level resolution [21,55], but can present challenges for characterizing novel divergent eukaryotic lineages due to high sequence divergence from existing database entries. The eKOI database comprises sequences for the entire COI gene for nearly all known eukaryotic phyla (Fig. 1), but lacks some phyla, like Dimorpha, Hemimastigophora, or Telonemia. Therefore, we propose that combining these independent nuclear and mitochondrial molecular markers could be ideal to uncover hidden patterns that may not be detected when relying on a single marker alone. Integrating mitochondrial eKOI (COI) and nuclear PR^2^ (18S) databases will provide new perspectives for hypothesis testing using eDNA.

Another key aim of eKOI is to facilitate the development of taxonomic-specific primers, similar to what PR^2^ [56] allows for 18S. This will allow applications of metabarcoding to reach beyond community-level analyses, enabling targeted protocols for specific taxonomic groups. Two examples are the use of COI metabarcoding for Arcellinida [33] and Foraminifera [11]. This enables applied studies, such as the development of bioindicators [57] and ecological assessments [22], to be focused on specific taxonomic groups using the COI gene. Furthermore, the integration of eKOI with eDNA-derived ASVs could offer a valuable tool for inferring biogeographical, diversification, or ecological patterns in future metabarcoding studies. While Fig. 4 illustrates this potential, the current sample size is too limited for robust inferences. These examples illustrate the potential of developing specific metabarcoding protocols targeting understudied protist groups, often hindered by sampling difficulties or the impossibility to culture some lineages.

Overall, the eKOI database represents a significant advancement for COI-based metabarcoding, particularly in the realm of protist diversity. eKOI provides a curated, comprehensive, and eukaryote-wide resource with a focus on previously underrepresented eukaryotic groups, not only addressing limitations of existing COI resources biased towards metazoans but also facilitating direct comparisons with 18S rRNA datasets. This integration paves the way for a deeper understanding of eukaryotic community structure and function in environmental samples. The potential for developing targeted primers and uncovering hidden biogeographical and ecological patterns further solidifies eKOI as a valuable tool for future research in protist diversity and ecology. Moreover, the integration of eKOI with the next version of the widely used PR^2^ database will enhance its long-term viability and facilitate regular updates, thereby increasing its applicability in future studies.

## Supplementary Material

baaf057_Supplemental_File

## Data Availability

The resulting eKOI taxonomic database, scripts, and raw data, such as alignments and phylogenetic trees generated in this study have been deposited in GitHub (https://github.com/rubenmiguens/eKOI_taxonomy_database.git) and figshare (10.1101/2024.12.05.626972), in the supplementary materials and PR^2^ database.
