# Supplementary figures and images for: A novel taxonomic database for eukaryotic mitochondrial cytochrome oxidase subunit I gene (eKOI), with a focus on protists diversity
Source: Database (Oxford). 2025 Sep 24;2025:baaf057. doi: 10.1093/database/baaf057 (PMC12462617; doi:10.1093/database/baaf057)

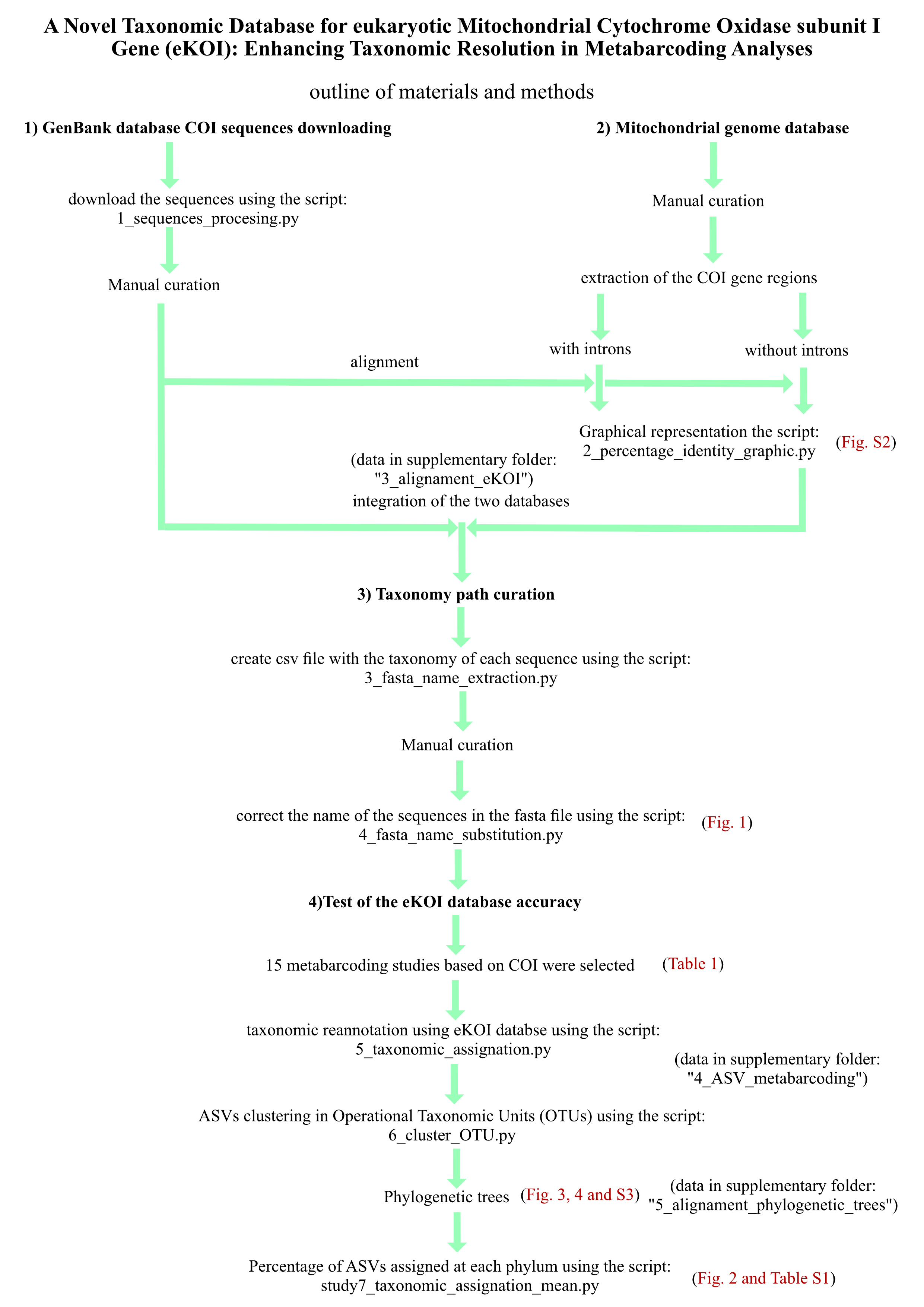

Supplement: baaf057_Supplemental_File [file baaf057_supplemental_file.zip › figure_s1.jpg]

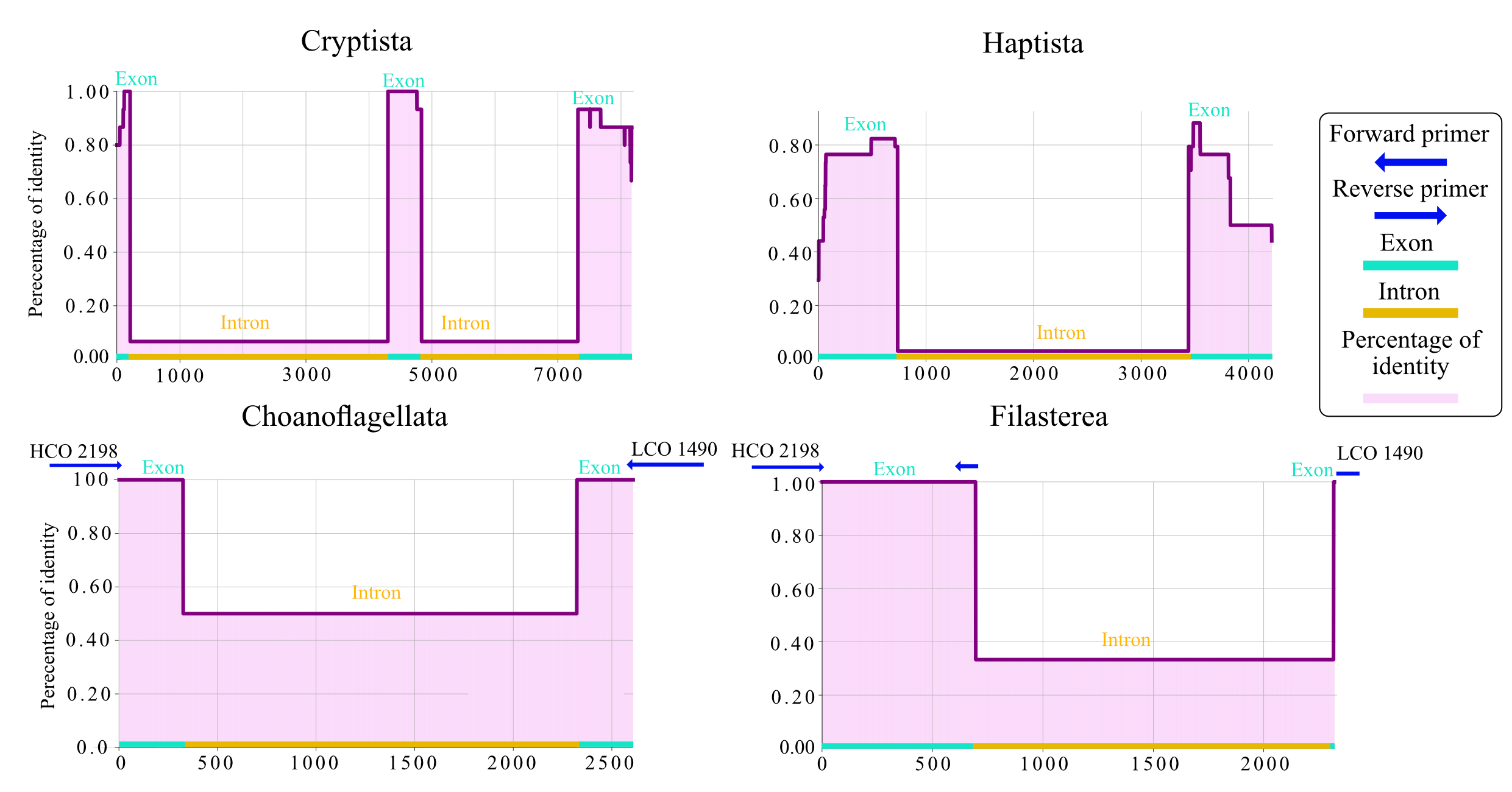

Supplement: baaf057_Supplemental_File [file baaf057_supplemental_file.zip › figure_s2.jpg]

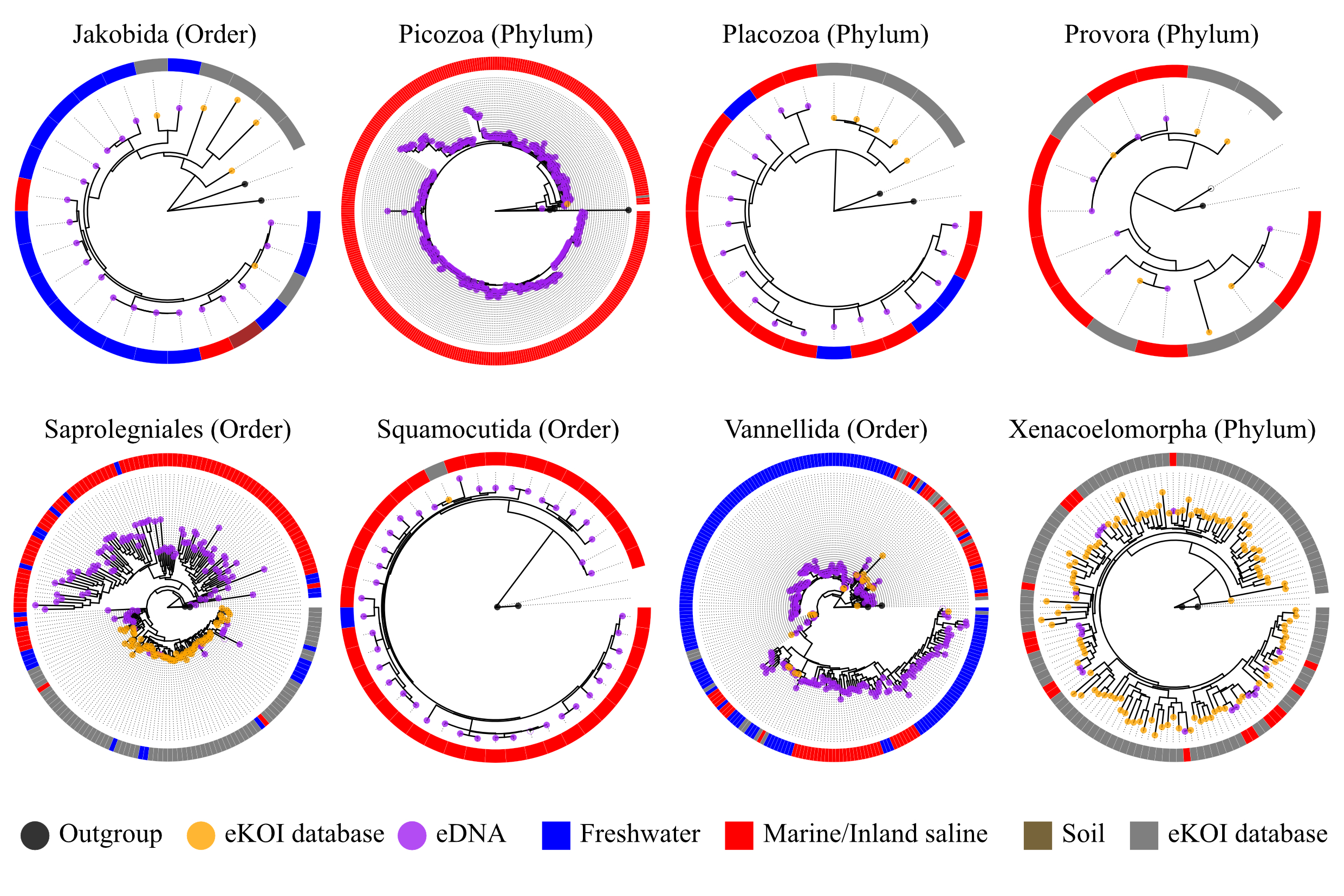

Supplement: baaf057_Supplemental_File [file baaf057_supplemental_file.zip › Figure_s3.jpg]

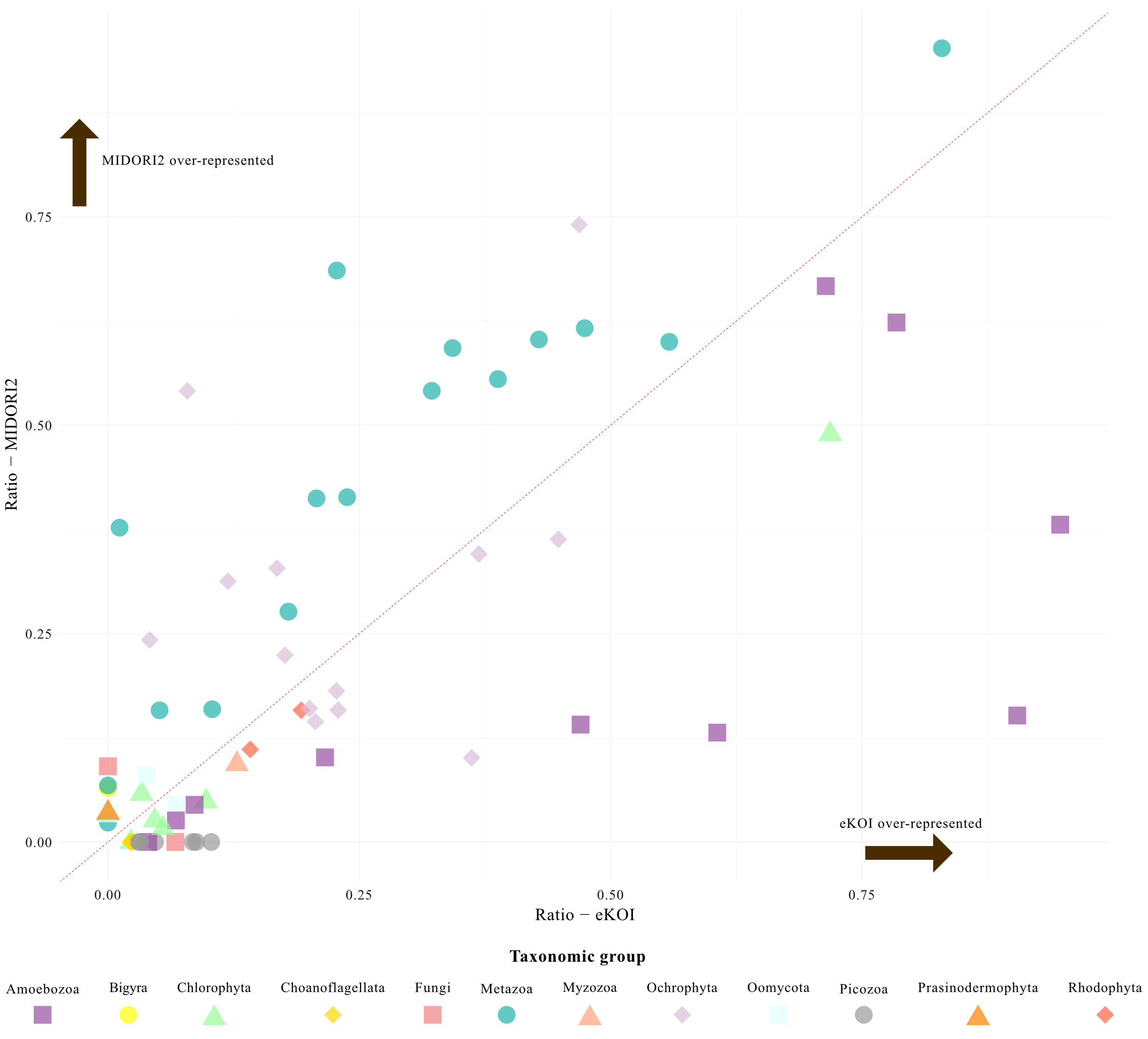

Supplement: baaf057_Supplemental_File [file baaf057_supplemental_file.zip › Figure_s4.jpg]

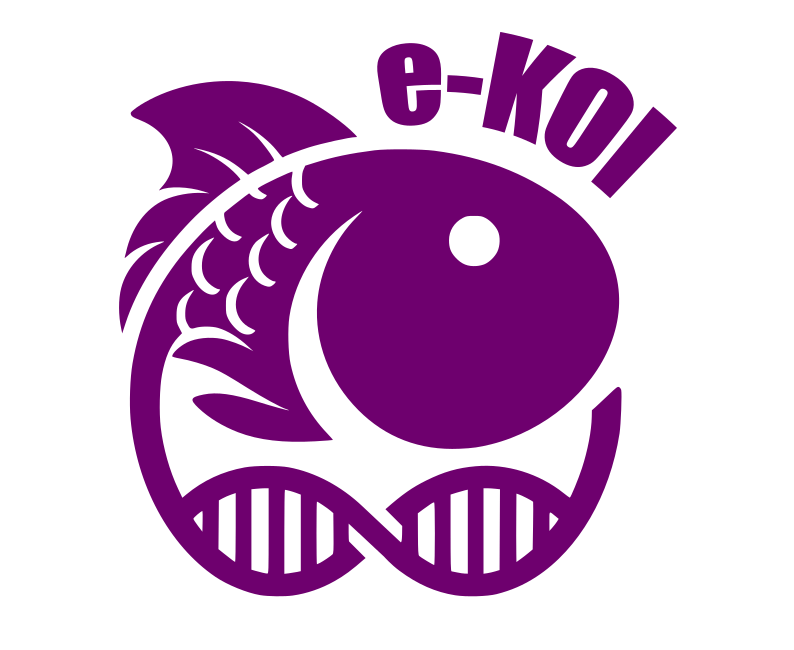

Supplement: baaf057_Supplemental_File [file baaf057_supplemental_file.zip › logo.png]

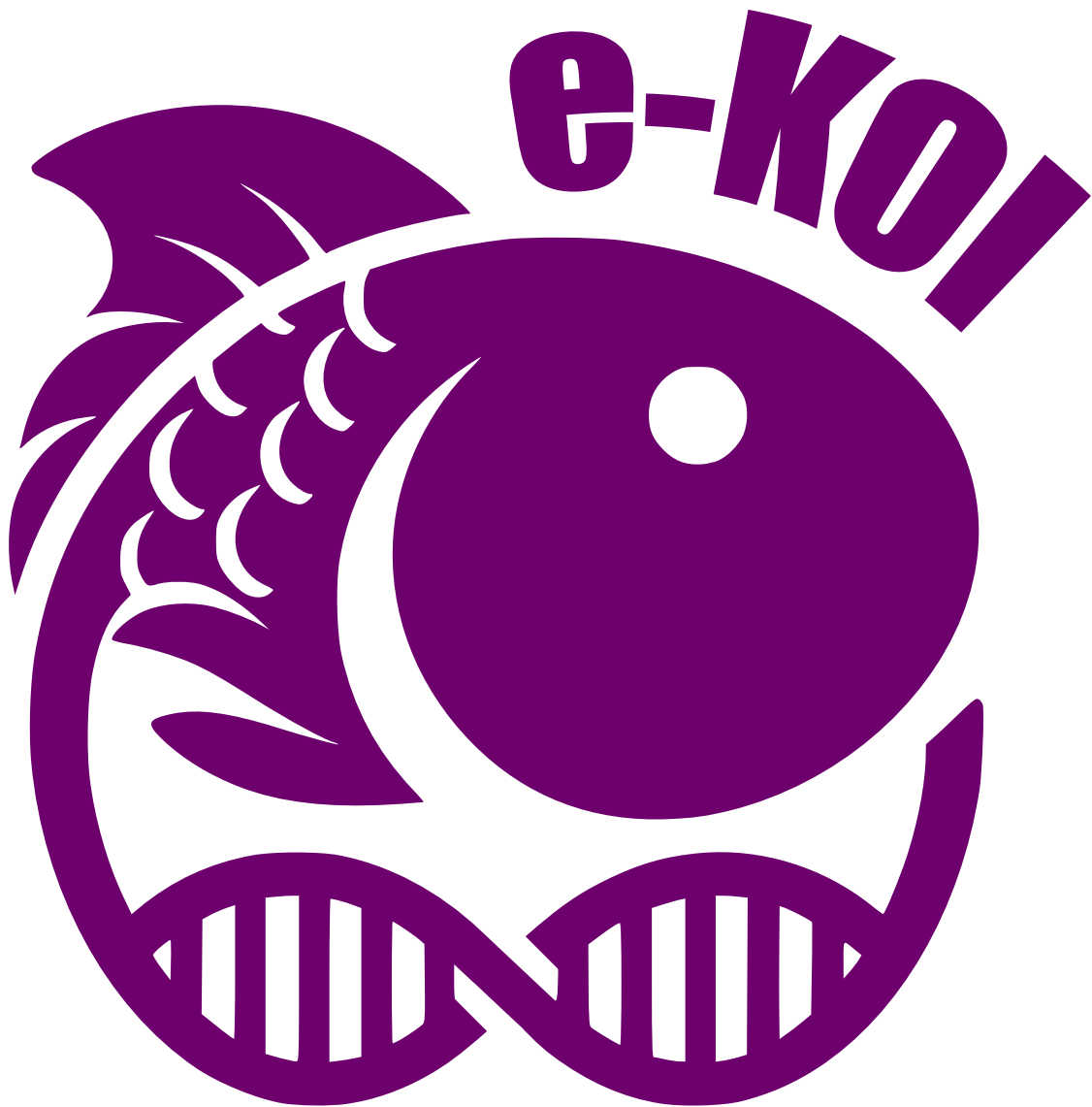

Supplement: baaf057_Supplemental_File [file baaf057_supplemental_file.zip › logo_purple.pdf]
